# Supplementary material for: Newly deceased Caribbean reef-building corals experience rapid carbonate loss and colonization by endolithic organisms
Source: Commun Biol. 2023 Sep 12;6:934. doi: 10.1038/s42003-023-05301-3 (PMC10497637; doi:10.1038/s42003-023-05301-3)
Supplement: Supplementary file 2 — Supplementary Information [file 42003_2023_5301_MOESM2_ESM.pdf]

Supplementary information

Supplementary Figure 1

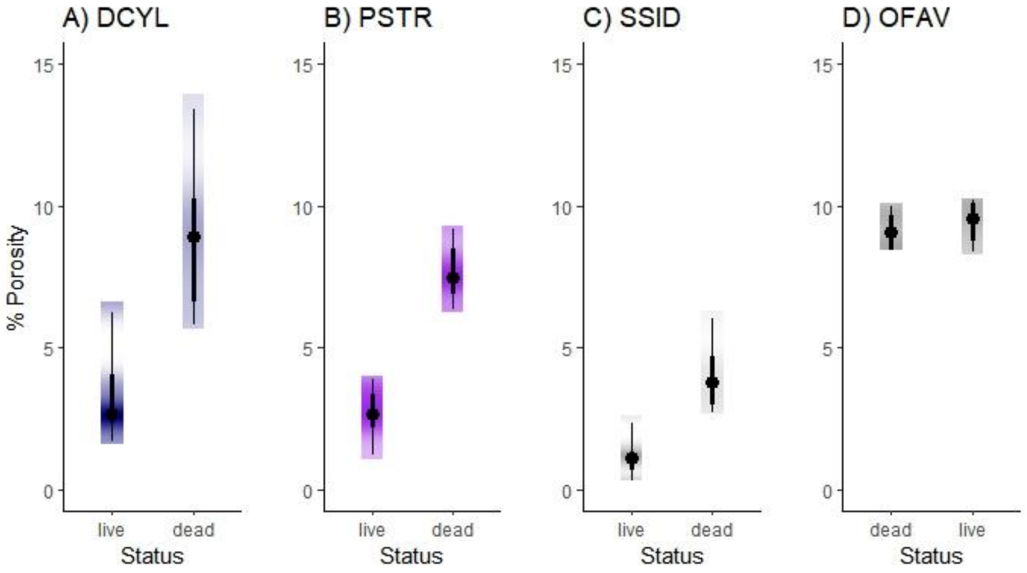

Supplementary Figure 1. Change in porosity in live vs dead colonies. The percent porosity of the three species was significantly different between live and dead colonies as shown in **a)** *Dendrogyra cylindrus*, **b)** *Pseudodiploria strigose*, and **c)** *Siderestraea siderea*. All live and dead slabs of all species were grouped together because there is only one value per slab. In all plots, the dark points inside the boxes represent the median of the distribution. The bold line represents interquartile range, and the thinner line represents the 95% CIs. The colors on the box indicate the distribution of data, brighter areas represent higher data density.

Supplementary Figure 2.

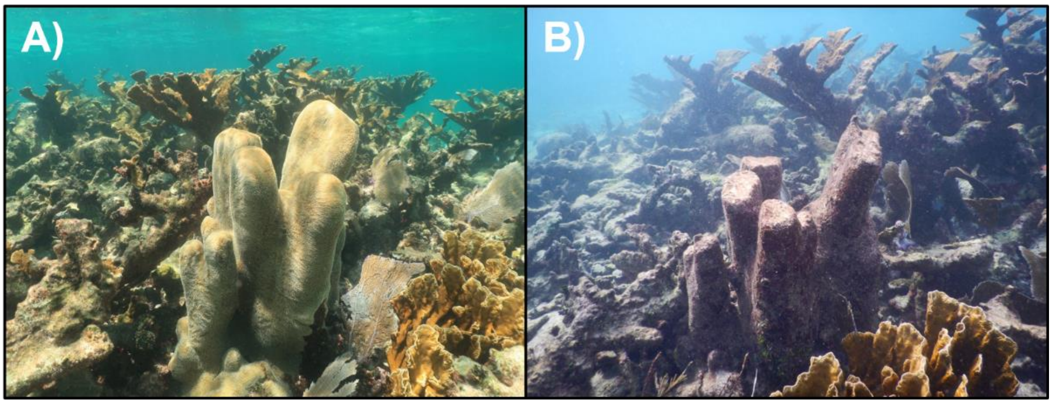

Supplementary Figure 2. Fragmentation process after dead skeletons are weakened by dissolution. a) Living and healthy colony before the stony coral tissue loss disease outbreak. **B)** Colony one year after its death. It is possible to observe the fragmentation of its pillars due to skeletal weakening.

Supplementary Figure 3

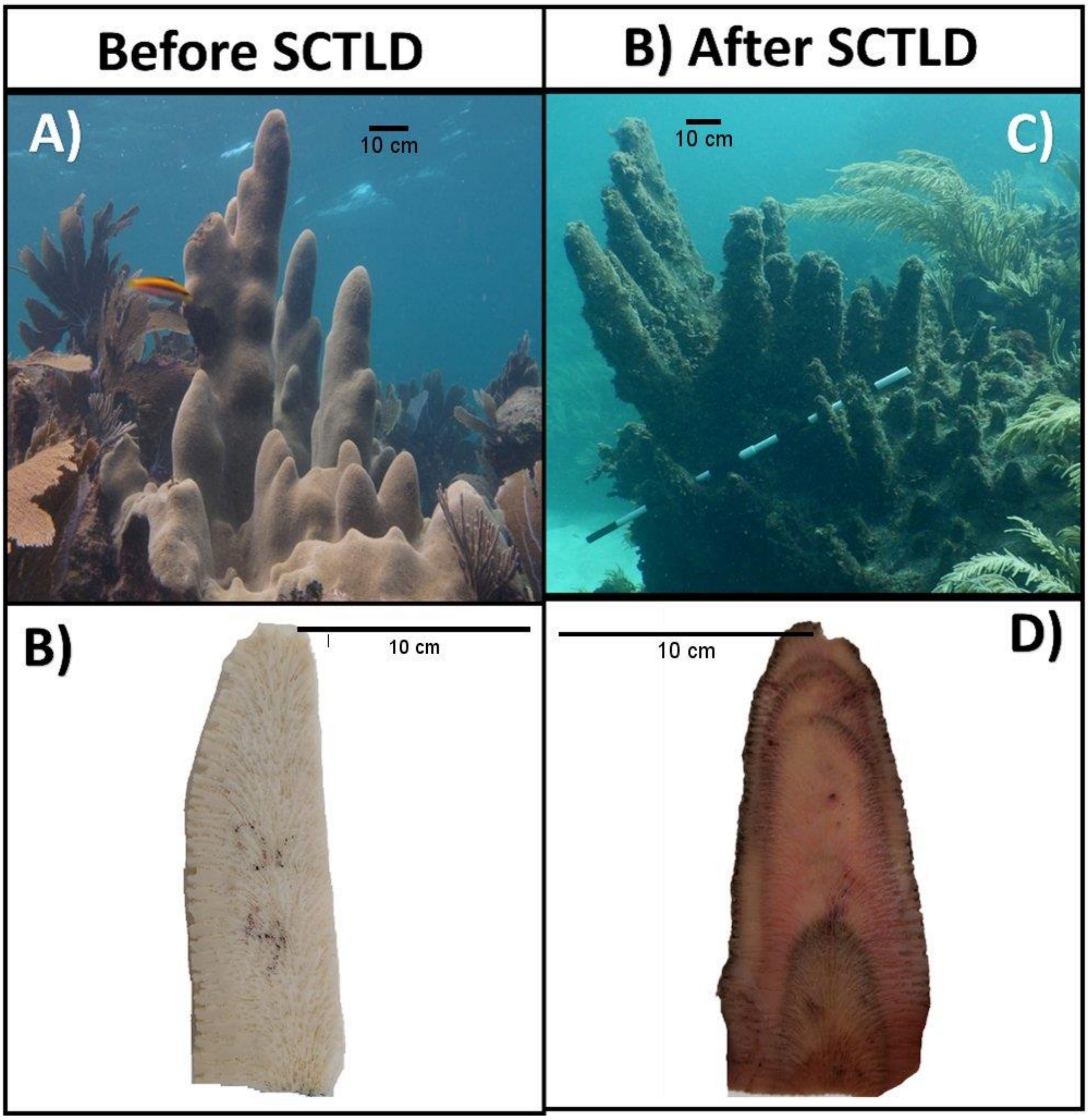

Supplementary Figure 3. Colonization process of epilithic and endolithic communities in a *Dendrogyra cylindrus* colony that had recently died. **a)** Live and healthy colony before the stony coral tissue loss disease outbreak. **b)** Slab obtained from a healthy colony. **c)** Colonization of algal mats, fleshy algae, and cyanobacteria in a recently dead *D. cylindrus* colony. D) Slab obtained from a dead colony.

Supplementary Figure 4.

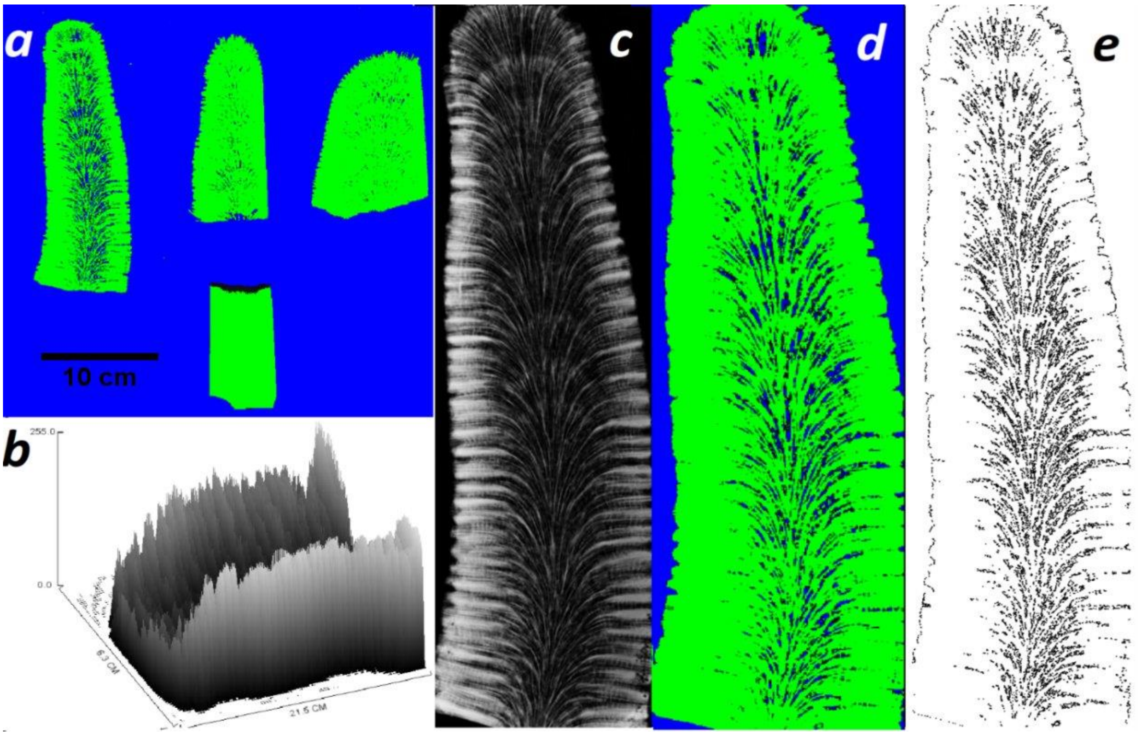

Supplementary Figure 4. Porosity analysis of coral slabs. Segmentation process in slabs of *Dendrogyra cylindrus*. **a**) Digital X-ray image segmented by thresholding. Blue corresponds to pixel values of 0–2 in grayscale (0–255), while green corresponds to values > 10. Slabs from left to right: CatedralC = Dead, Catedral5 = Live, Catedral6 = Live. Note the visible difference in porosity of the dead slab compared to those of the live slabs. **b**) Value of each pixel (0–255) throughout the slab (example slice CatedralC). **c)** CatedralC slab segmented by thresholding. **d)** Green corresponds to solid $CaCO_3$ structures, and blue corresponds to the porosity spaces. The black areas are transition regions between one threshold and another. **e)** Image showing the porous regions.

Supplementary Figure 5.

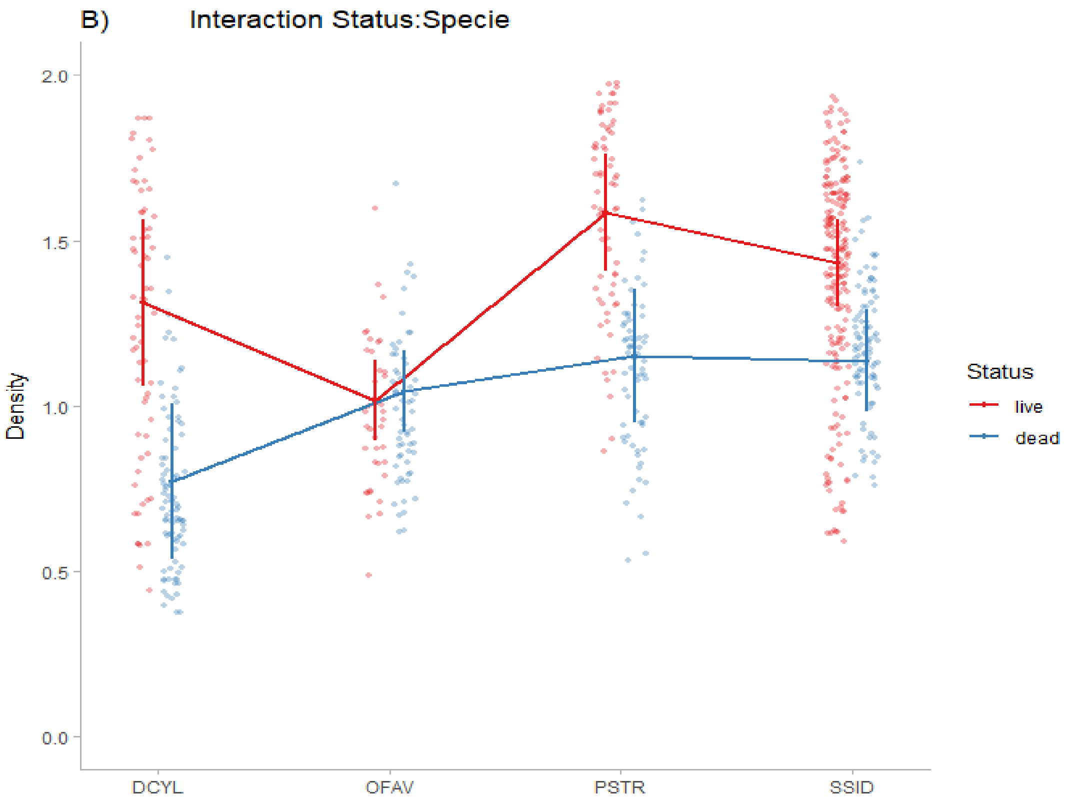

Supplementary Figure 5. Change in density between live vs dead colonies according to the General Model 1. *lmer(Density~Specie*Status+Year*Status+(1|Zone)+(1+Specie|ID)+(1|Extension).* Interaction between Specie and Status – to test change density between live vs dead colonies. Absolute mass change between live vs dead colonies in each specie. The dots indicate the annual density values calculated for each species according to its state (i.e., live or dead). The vertical lines above the dots indicate the 95% CI (for more details see supplementary tables 14-16).

Supplementary Figure 6.

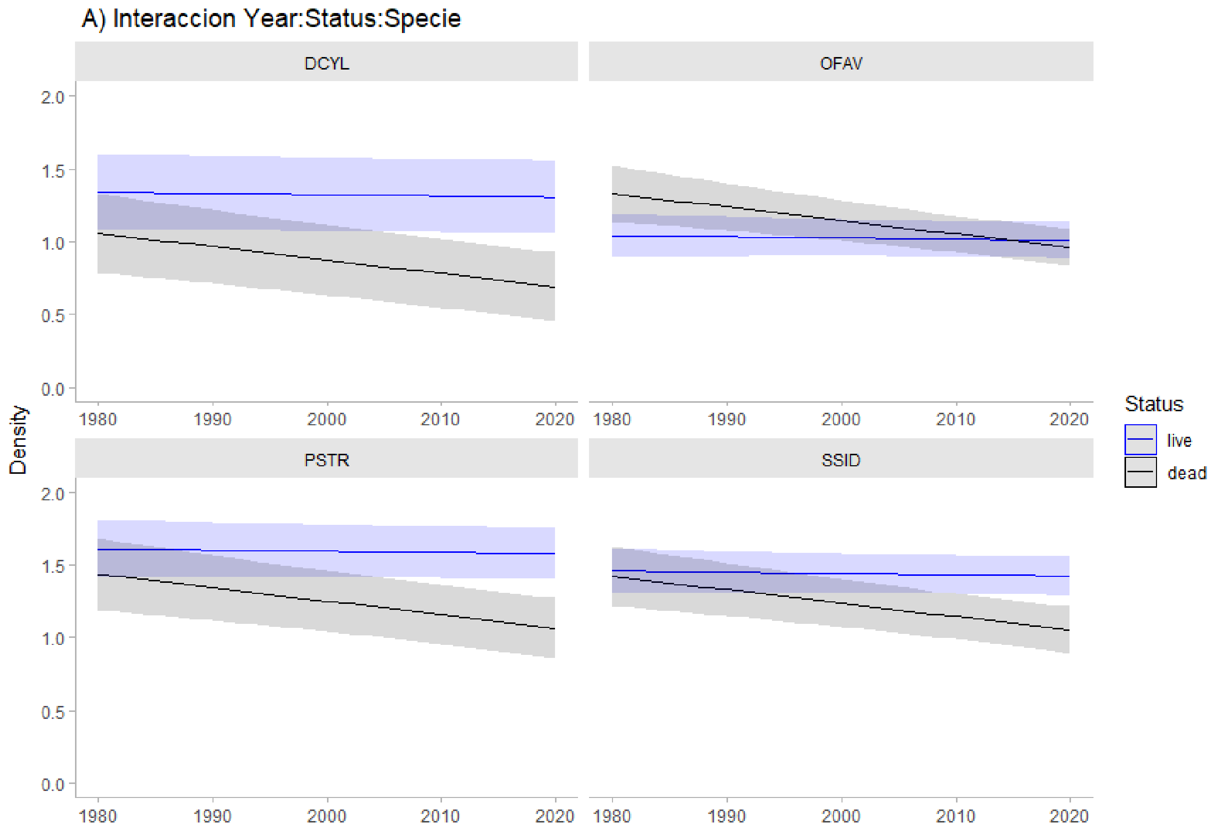

Supplementary Figure 6. Change in density across skeletal sections between live vs dead colonies according to the General Model 1.
*lmer(Density~Specie*Status+Year*Status+(1|Zone)+(1+Specie|ID)+(1|Extension).*
Interaction Year and Status – to test change density across the colonies. Trends for each species in density change in different sections of the colonies (current years refer to shallow sections of the colony, older years indicate deeper sections of the skeleton). Shading represents the 95% CI (for more details see supplementary tables 14-16)

Supplementary Figure 7.

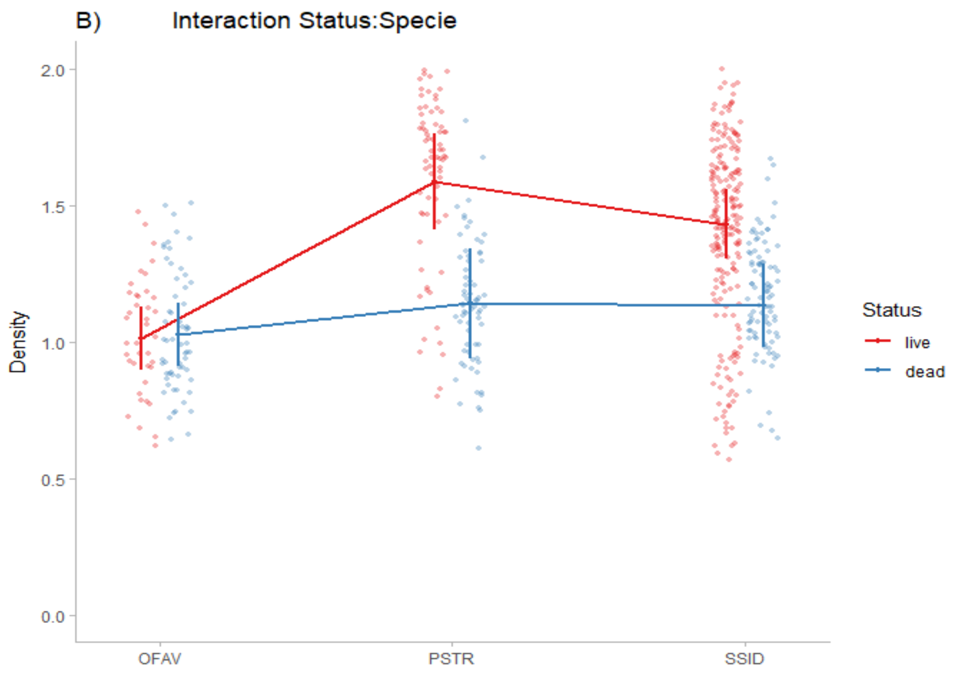

Supplementary Figure 7. Change in density between live vs dead colonies according to the General Model 1 *without *D. cylindrus* data. Interaction between Specie and Status – to test change density between live vs dead colonies. Absolute mass change between live vs dead colonies in each specie. The dots indicate the annual density values calculated for each species according to its state (i.e., live or dead). The vertical lines above the dots indicate the 95% CI (for more details see supplementary tables 17-19)

Supplementary Figure 8.

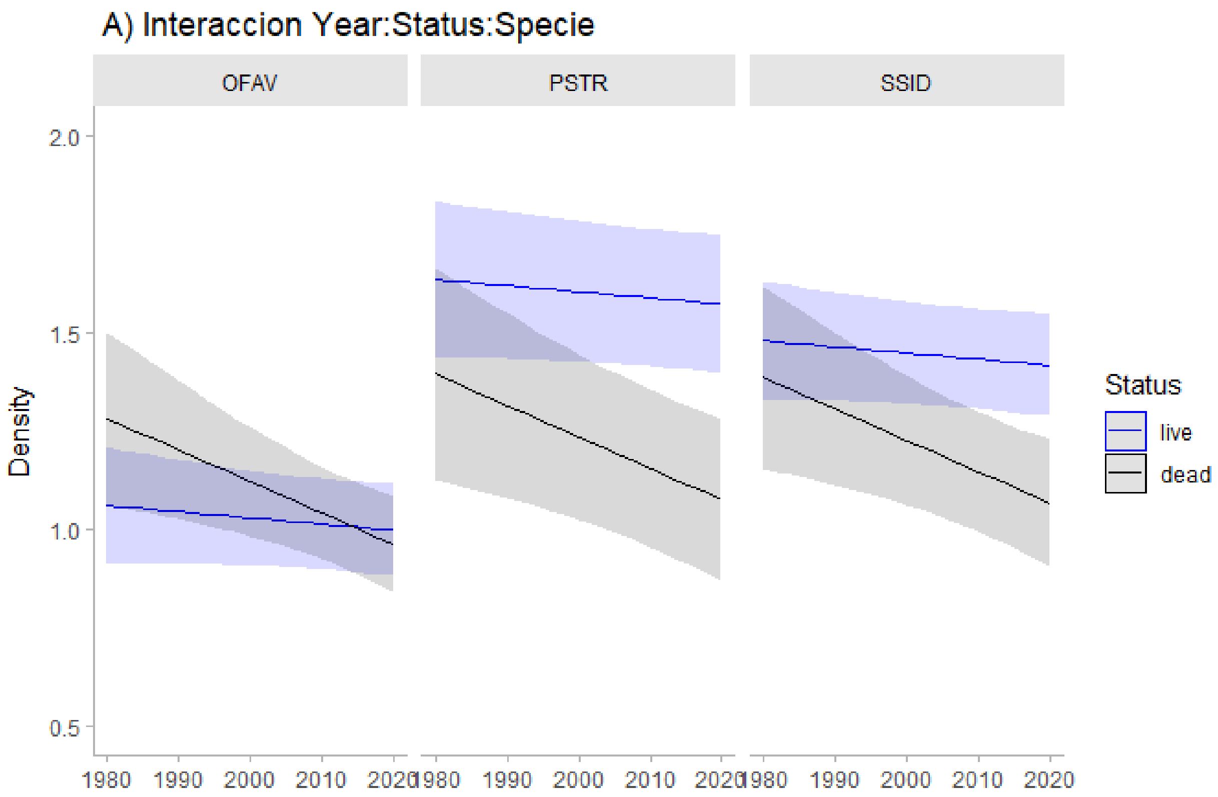

Supplementary Figure 8. Change in density across skeletal sections between live vs dead colonies according to the General Model 1 *without *D. cylindrus* data. Interaction Year and Status – to test change density across the colonies. Trends for each species in density change in different sections of the colonies (current years refer to shallow sections of the colony, older years indicate deeper sections of the skeleton). Shading represents the 95% CI (for more details see supplementary tables 17-19)

Supplementary Table 1. Summary of density data for each core of *Dendrogyra cylindrus*, *Pseudodiploria strigosa, Siderastrea siderea,* and *Orbicella faveolata.* Values are the mean (± SE) of all annual skeletal density measurements per core. Years is number of years of data obtained from each core.

Species	CoreID	Yrs	Status
D. cylindrus			
	D1 LC	16	dead_1yr
	D2 LC	12	dead_1yr
	D3 LC	21	dead_1yr
	D4 LC	11	dead_2yr
	D5 LC	10	live
	D6 LC	11	live
	D7 LC	11	live
	D8 LC	10	live
	D1 LP	11	dead_2yr
	D2 LP	12	live
	D1 RP	17	dead_2yr
	D2 RP	15	live
P. strigosa			
	P1 BR	6	live
	P2 BR	11	live
	P3 BR	7	live
	P4 BR	14	live
	P5 BR	15	dead
	P6 BR	15	dead
	P7 BR	15	dead
	P8 FR	13	live
	P9 FR	20	live
	P10FR	7	live
	P11FR	14	dead
	P12FR	7	dead
S. siderea			
	S1 BR	14	live
	S2 BR	11	live
	S3 BR	14	live
	S4 BR	6	live
	S5 BR	32	live
	S6 BR	14	live
	S7 BR	22	dead
	S8 BR	22	dead
	S9 BR	6	dead
	S10FR	21	live
	S11FR	38	live
	S12FR	36	live
	S13FR	6	live
	S14FR	15	dead
	S15FR	15	dead

	S16 BR	15	dead
	S17 BR	6	dead
O. faveolate			
	O1 BR	14	live
	O2 BR	5	live
	O3 BR	9	live
	O4 BR	11	live
	O5 BR	7	dead
	O6 BR	13	dead
	O7 BR	13	dead
	O8 BR	8	dead
	O9 BR	7	dead
	O10 BR	12	dead

Supplementary Table 2. Percentage loss of coral skeletal density in *Dendrogyra cylindrus, Siderestraea siderea*, *Pseudodiploria strigose*, and *Orbicella faveolate*. The growth type and structural morphology of each species is highlighted.

**Species**	**Density live (g·$CaCO_3$ cm^{-3})**	**Density dead (g·$CaCO_3$ cm^{-3})**	**Lost Density (%)**	**Structural morphology**	**Growth type**
D. cylindrus	1.31 ± 0.03	0.74 ± 0.05	-42.97%	Meandroid	Columnar
P. strigosa	1.58 ± 0.02	1.14 ± 0.02	-31.20%	Meandroid	Pseudo-masive
S. siderea	1.42 ± 0.03	1.18 ± 0.08	-15.71%	Cerioid	Pseudo-masive
O. faveolata	1.08 ± 0.03	0.99 ± 0.02	-1.70%*****	Plocoid	Masive

Supplementary Table 3. Main reported rates of microbioerosion. *For the purposes of comparison, estimates for Reyes-Nivia et al. (2013) and Leggat et al. (2019) were transformed from mg $CaCO_3$ cm^{-2} $month^{-1}$ and g $CaCO_3$ m^{-2} $month^{-1}$ to kg $CaCO_3$ m^{-2} $year^{-1}$ respectively.

Reference	Substrate	Data source	Time deployment	Number of replicates	Approach to measure micro erosion	kg $CaCO_3$ m^{-2} $year^{-1}$
Grange et al.[1]	Coral blocks (*Porites* sp.)	*In situ*	1–12 months	Three per month Total=24	SEM	0.93 ± 0.04
Leggat et al.[2]	Fragments (*Pocillopora damicornis* and *Acropora aspera*)	Experimental	1–2 months	Two per specie, tank and treatment Total= 16**	Porosity analysis of micro-CT scans	19.00*
Enochs et al. [3]	Coral blocks (*Porites* sp.)	*In situ*	2 years	35 per site Total= 70	Optical densitometry of micro-CT scans	1.52 ± 0.08
Enochs et al. [4]	Coral blocks (*Porites* sp.)	*In situ*	3–4 months	10 for three coral reef site Total= 30	Optical densitometry of CT scans	NA**
Enochs et al. [5]	Calcite blocks	*In situ*	1.8 years	eight for three coral reef site Total= 24	Image analysis	0.46 ± 0.02
Reyes-Nivia et al. [6]	Colonies (Porites cylindrica and Isopora cuneata)	Experimental	1 month	45 per specie Total= 90	buoyant weight	4.92 ± 0.3*
Tribollet and Golubic. [7]	Coral blocks (*Porites* sp.)	*In situ*	1–3 years	Four per year Total= 12	SEM	1.35 ± 0.5
Tribollet et al. [8]	Coral blocks (*Porites* sp.)	*In situ*	1 year	Four per site and year Total= 20	SEM	1.34 ± 0.74
Chazottes et al. [9]	Coral blocks (*Porites lobata*)	*In situ*	1 year	Three per station, Total=24	SEM	0.01 ± 0.04
Mallela and Perry et al. [10]	Recently dead colonies of seven species	*In situ*	NA	Agaricia agaricites = 25 Agaricia lamarcki = 4 Diploria strigosa = 1 Helioceris cucullata= 18 Meandrina meandrites= 2 Porites astreoides = 6 Siderastrea siderea = 3 Total= 59	SEM	0.17
Vogel et al.[11]	Differetnts experimental substrates	*In situ*	3–24 months	12 per period Total=80	SEM	0.27
Roik et al.[12]	Limestone blocks	*In situ*	6, 12 and 30 months	12 per period Total=36	Dry weigth	0.61-0.96 Net erosion
Brown et al.[13]	Coral blocks (*Porites* sp.)	*In situ*	1 year	Eigth per site Total= 56	buoyant weight	0.001
			**Averege of treatment**	**11.8**		
			**Averege of replicates**	**41.61**		

Supplementary Table 4. Area of each reef unit in the Puerto Morelos reef system and number of transects used to survey each reef unit. These areas were obtained from ArrecifeSAM-CONABIO[14].

Reef unit	Total area (m^2)
Bonanza	349629.4
Tanchacte	682125.49
Limones	343293.76
Puerto Morelos	1452870.76
La Pared	164087.51
Jardines	153960.23
Puerto Morelos reef system	3145967.15

Supplementary Methods

Appendix 1. Water chemistry

In order to evaluate both pH and the aragonite saturation state of the seawater of the reef where coral skeleton cores were obtained (i.e., La Catedral), monthly discrete samples were collected between November 2020 and March 2021. Discrete samples were obtained around noon using a Niskin bottle, from which 500 mL was transferred to borosillicate glass bottles and immediately poisoned with 100 µL saturated mercury chloride ($HgCl_2$) solution[15]. Salinity was measured with a YSI 3100 probe (± 0.1 PSU). In the lab, the dissolved inorganic carbon (DIC) concentration was analysed by coulometry with an error ≤ 3.0 µmol kg^{-1} [15]. Total alkalinity (TA) was determined by acidic titration using an open cell [15] with an of error ≤ 3.0 µmol kg^{-1}. Certified reference material (AG Dickson, Scripps Institution of Oceanography) was used to ensure accurate readings within ≤ 0.15% for DIC and TA. The pH (seawater scale; PHSW) and aragonite saturation state were calculated using in situ temperature, salinity, DIC, and TA values with CO_2sys software [16].

Sensor data and discrete water samples were collected periodically from the reefs where the coral skeleton samples were obtained. The dissolved inorganic carbon (DIC), total alkalinity (TA), temperature, pH, aragonite saturation sate (Ω_{arag}), and partial pressure of CO_2 (pCO_2) of the water surrounding dead skeletons and live corals indicated that the water was neither acidifying nor warming (Table 2). The average DIC was 2035 ± 7.614 µmol kg^{-1} and the average TA was 2343 ± 4.662 µmol kg^{-1}. The average temperature was 28.06 °C, and temperatures fluctuated between 26.34 °C and 29.72 °C. The pH reached maximum and minimum values of 8.16 and 7.86 pH units, respectively. The lowest pH values were consistently recorded during the night. The average Ω_{arag} was 3.521 ± 0.088. Finally, the average pCO_2 value was 451± 16.389.

Supplementary Table 5. Means for dissolved inorganic carbon (DIC), total alkalinity (TA), temperature, pH, aragonite saturation sate (Ω_{arag}), and partial pressure of CO_2 (pCO_2). Chemistry parameters of water samples collected from November 2020 to March 2021 at La Catedral. Data are mean ± SE. In March 2021, we collected 4 water samples.

Date	DIC (µmol·kg^{-1})	TA (µmol·kg^{-1})	pH *in situ*	Ω_{arag}	pCO_2
nov-2020	2034	2327	7.98	3.36	484
dic-2020	2031	2358	8.04	3.73	417
feb-2021	2039	2346	8.00	3.51	453
mar-2021	2035 ± 10.68	2343 ± 4.48	8.00 ± 0.01	3.51 ± 0.11	450.75±20.73

Appendix 2. Model selection diagnostics – Linear mixed effects

Colony identity (ID), reef area (back-reef or fore-reef), and the annual extension rate (i.e., extension between growth bands across each core) were alternated in various models as nested or orthogonal fixed or random effects according to different hypotheses. For the selection of the best model, we used the information from the Akaike Information Criterion (AIC), Bayesian information criterion (BIC), and the marginal r^2 using the "glance" function of the "jtools" package. The goodness of fit of each LMM was evaluated with the *DHARMa* package via an analysis of residuals.

(1) Fitting models of *Dendrogyra cylindrus*

M1D: lmer(Density~Year*Status+Reef+(1|ID)+(1|Extension))
M2D: lmer(Density~Year*Status+(1|Reef)+(1|ID)+(1|Extension))
M3D: lmer(Density~Year*Status+(1|Reef)+(1+Status|ID)+(1|Extension))
M4D: lmer(Density~Year*Status+Reef+(1+Status|ID)+(1|Extension))

M1D: Random slopes:Reef fixed
M2D: Random slopes:Reef random
M3D: Random slopes and intercepts:Reef random
M4D: Random slopes and intercepts:Reef fixed

*In all models status and Year:Status interaction was significance. While variable Reef was not significance when it considers like fixed factor.

Comparison of Akaike Information Criterion (AIC), and Bayesian Information Criterion (BIC) scores to select the best model by *D. cylindrus*. Lower AIC and BIC values indicate better models. R2 marginal and R2 conditional explain the variance of the fixed effects and the variance of the complete model respectively.

	AIC	BIC	R2(marg)	R2(cond)
M1D:	-61.376	-27.618	0.567	0.890
**M2D:**	**-61.577**	**-30.888**	**0.384**	**0.905**
M3D:	-58.924	-12.890	0.777	0.905
M4D:	-58.820	-9.718	0.561	0.893

Supplementary Table 6. Analysis of Deviance Table (Type II Wald chi-square tests) of the Model M2D testing the significance of three different variables on skeletal density changes.

Response: skeletal density

	**Chisq**	**df**	**Pr(>Chisq)**
Year	4.7473	1	0.0293444 *
Status	14.0718	2	0.0008797 ***
Year:Status	19.4012	2	6.125e-05 ***

Supplementary Table 7. Parameter estimates from the Model M2D were used to test the effect of three different effects Year, Status, and interaction between Year and Status. Random effects are shown as standard deviations.

**Predictor**	**Estimate**	**Std. Error**	**t value**
Fixed effects			
(Intercept)	-22.349	10.277	-2.175
Year	0.011	0.005	2.300
Status [dead_1yr]	55.765	12.831	4.346
Status [dead_2yr]	32.709	15.905	2.056
Year × Status [dead_1yr]	-0.028	0.006	-4.399
Year × Status [dead_2yr]	-0.016	0.007	-2.082

Random effects	**Name**	**Variance**	**Std. Dev.**
Extension	(Intercept)	1.227e-10	1.108e-05
ID	(Intercept)	6.485e-02	2.547e-01
Reef	(Intercept)	5.489e-02	2.343e-01
Residual		2.189e-02	1.480e-01

(2) Fitting models of *Pseudodiploria strigosa*, and *Siderastrea siderea* (same model structure for both species)

M1: lmer(Density~Year*Status+Zone+(1|ID)+(1|Extension))
M2: lmer(Density~Year*Status+(1|Zone)+(1|ID)+(1|Extension))
M3: lmer(Density~Year*Status+(1|Zone)+(1+Status|ID)+(1|Extension))
M4: lmer(Density~Year*Status+Zone+(1+Status|ID)+(1|Extension))

M1: Random slopes:Zone fixed
M2: Random slopes:Zone random
M3: Random slopes and intercepts:Zone random
M4: Random slopes and intercepts:Zone fixed

*In all models status and Year:Status interaction was significance. While variable Zone was not significance when it consider like fixed factor.

Comparison of Akaike Information Criterion (AIC), and Bayesian Information Criterion (BIC) scores to select the best model by *P. strigosa*. Lower AIC and BIC values indicate better models. R2 marginal and R2 conditional explain the variance of the fixed effects and the variance of the complete model respectively.

	AIC	BIC	R2(marg)	R2(cond)
M1P:	-30.631	-7.041	0.465	1.000
**M2P**:	**-30.756**	**-7.166**	0.334	1.000
M3P:	-36.283	-6.795	0.999	NA
M4P:	-32.027	-2.539	0.375	1.000

Supplementary Table 8. Analysis of Deviance Table (Type II Wald chi-square tests) of the Model 2P testing the significance of three different variables on skeletal density changes.

Response: skeletal density

	Chisq	df	Pr(>Chisq)
Year	5.6835	1	2.02e-16***
Status	6.0216	1	0.0009812 ***
Year:Status	6.9933	1	2.02e-16 ***

Supplementary Table 9. Parameter estimates from the Model M3P were used to test the effect of three different effects Year, Status, and interaction between Year and Status. Random effects are shown as standard deviations.

Predictor	Estimate	Std. Error	t value
Fixed effects			
(Intercept)	-1.53e+01	6.955e-01	-22.004
Year	8.38e-03	3.397e-04	24.697
Status [live]	2.45e+01	2.734e+00	8.977
Year × Status [live]	-1.24e-02	1.358e-03	-9.139
Random effects	**Name**	**Variance**	**Std. Dev.**
Extension	(Intercept)	3.018e-02	1.737e-01
ID	(Intercept)	5.489e-11	7.409e-06
Zone	(Intercept)	0.000e+00	0.000e+00
Residual		3.106e-05	5.573e-03

Comparison of Akaike Information Criterion (AIC), and Bayesian Information Criterion (BIC) scores to select the best model by *S. siderea*. Lower AIC and BIC values indicate better models. R2 marginal and R2 conditional explain the variance of the fixed effects and the variance of the complete model respectively.

	AIC	BIC	R2(marg)	R2(cond)
M1S:	-38.751	-8.730	0.167	0.712
**M2S:**	**-41.272**	**-11.252**	**0.159**	**0.701**
M3S:	-39.422	-1.896	0.156	0.711
M4S:	-37.654	-0.129	0.192	0.724

Supplementary Table 10. Analysis of Deviance Table (Type II Wald chi-square tests) of the Model 2S testing the significance of three different variables on skeletal density changes.

Response: skeletal density

	Chisq	df	Pr(>Chisq)
Year	5.6835	1	0.017125 *
Status	6.0216	1	0.014132 *
Year:Status	6.9933	1	0.008181 **

Supplementary Table 11. Parameter estimates from the Model M2S were used to test the effect of three different effects Year, Status, and interaction between Year and Status. Random effects are shown as standard deviations.

**Predictor**	**Estimate**	**Std. Error**	**t value**
Fixed effects			
(Intercept)	5.361	3.174	1.689
Year	-0.001	0.001	-1.238
Status [live]	22.499	8.604	2.615
Year × Status [live]	-0.011	0.004	-2.644
Random effects	**Name**	**Variance**	**Std. Dev.**
Extension	(Intercept)	1.249e-02	1.118e-01
ID	(Intercept)	3.814e-02	1.953e-01
Zone	(Intercept)	3.437e-11	5.863e-06
Residual		2.790e-02	1.670e-01

(3) Fitting models of *Orbicella faveolata* (only collected in back-reef)

M1O: lmer(Density~Year*Status+(1|ID)+(1|Extension))
M2O: lmer(Density~Year*Status+(1+Status|ID)+(1|Extension))

M1O: Random slopes
M2O: Random slopes and intercepts

Comparison of Akaike Information Criterion (AIC), and Bayesian Information Criterion (BIC) scores to select the best model by *O. faveolate*. Lower AIC and BIC values indicate better models. R2 marginal and R2 conditional explain the variance of the fixed effects and the variance of the complete model respectively.

	AIC	BIC	R2(marg)	R2(cond)
**M1O:**	**-18.910**	**-0.674**	**0.005**	**0.311**
M2O:	-15.629	7.818	0.003	0.317

Supplementary Table 12. Analysis of Deviance Table (Type II Wald chi-square tests) of the Model 1O testing the significance of three different variables on skeletal density changes.

Response: skeletal density

	Chisq	df	Pr(>Chisq)

Year	0.3689	1	0.5436
Status	0.0098	1	0.9213
Year:Status	0.1911	1	0.6620

Supplementary Table 13. Parameter estimates from the Model M1O were used to test the effect of three different effects Year, Status, and interaction between Year and Status. Random effects are shown as standard deviations.

Predictor	Estimate	Std. Error	t value
Fixed effects			
(Intercept)	13.094	16.721	0.783
Year	-0.005	0.008	-0.723
Status	-9.451	21.604	-0.437
Year × Status	0.004	0.010	0.437
Random effects	**Name**	**Variance**	**Std. Dev.**
Extension	(Intercept)	0.001	0.034
ID	(Intercept)	0.011	0.106
Residual		0.028	0.168

General Model diagnostic.

(4) Fitting general model that include data of *Dendrogyra cylindrus, Psudodiploria strigosa, Siderastrea sidereal* and *Orbicella faveolate.*

M1G: lmer(Density~Specie*Status+Year*Status+(1|Zone)+(1+Specie|ID)+(1|Extension))
***M2G: lmer(Density~Specie*Status+Year*Status+(1|Zone)+(1+Specie|ID)+(1|Extension))**

M1G and M2G: Random intercepts and slopes: Interaction between Specie and Status – to test change density between live vs dead colonies. Interaction Year and Status – to test change density across de colonies.

*M2G without *D. cylindrus* data.
**These models have not been compared using AIC because M1G has more observations (*D. cylindrus* annual data) and therefore they are not comparable. However, they have the same structure (interactions, fixed factors and random factors).

Supplementary Table 14. Analysis of Deviance Table (Type II Wald chi-square tests) of the Model M1G testing the significance of three different variables on skeletal density changes.

Response: skeletal density

	Chisq	df	Pr(>Chisq)
Specie	31.4395	3	6.869e-07 ***
Status	13.1280	1	0.0002932 ***

Year	7.0126	1	0.0083623 **
Specie:Status	21.3256	3	9.009e-05 ***
Status:Year	10.2819	1	0.0013434 **

Supplementary Table 15. Parameter estimates from the Model M1G were used to test the effect of three different effects Year, Status, and interaction between Year and Status. Random effects are shown as standard deviations.

Predictor	Estimate	Std. Error	t value
Fixed effects			
(Intercept)	19.438	4.541	4.280
SpecieOFAV	0.272	0.136	2.002
SpeciePSTR	0.378	0.158	2.386
SpecieSSID	0.365	0.144	2.531
Statuslive	-16.443	5.297	-3.104
Year	-0.009	0.002	-4.111
SpecieOFAV:Statuslive	-0.569	0.184	-3.085
SpeciePSTR:Statuslive	-0.106	0.214	-0.495
SpecieSSID:Statuslive	-0.245	0.194	-1.259
Statuslive:Year	0.008	0.002	3.207
Random effects	**Name**	**Variance**	**Std. Dev.**
Extension	(Intercept)	0.007	0.088
ID	(Intercept)	0.089	0.298
	SpecieOFAV	0.093	0.305
	SpeciePSTR	0.096	0.310
	SpecieSSID	0.139	0.373
Zone	(Intercept)	0.002	0.044
Residual		0.028	0.168

Supplementary Table 16. MG1 Contrast test. Degrees-of-freedom method: kenward-roger. Confidence level used: 0.95

Contrast	Estimate	Std. Error	t.ratio	p.value
Specie =DCYL:				
Contrast dead-live	-0.530	0.171	-3.109	0.010
Specie= OFAV				

Contrast dead-live	0.039	0.124	0.319	0.766
Specie=PSTR				
Contrast dead-live	-0.424	0.130	-3.262	0.009
Specie=SSID				
Contrast dead-live	-0.285	0.133	-2.142	0.059

Supplementary Table 17. Analysis of Deviance Table (Type II Wald chi-square tests) of the Model M2G (without *D. cylindrus* data) testing the significance of three different variables on skeletal density changes.

Response: skeletal density

	**Chisq**	**df**	**Pr(>Chisq)**
Specie	32.0216	2	1.113e-07 ***
Status	7.4500	1	0.0063436 **
Year	4.9814	1	0.0256208 *
Specie:Status	15.6478	2	0.0004001 ***
Status:Year	4.0228	1	0.0448902 *

Supplementary Table 18. Parameter estimates from the Model M2G were used to test the effect of three different effects Year, Status, and interaction between Year and Status. Random effects are shown as standard deviations.

**Predictor**	**Estimate**	**Std. Error**	**t value**
Fixed effects			
(Intercept)	17.073	5.752	2.968
SpeciePSTR	0.113	0.111	1.014
SpecieSSID	0.104	0.092	1.131
Statuslive	-12.927	6.443	-2.006
Year	-0.007	0.002	-2.792
SpeciePSTR:Statuslive	0.461	0.148	3.109
SpecieSSID:Statuslive	0.315	0.103	3.057
Statuslive:Year	0.006	0.003	2.006
Random effects	**Name**	**Variance**	**Std. Dev.**
Extension	(Intercept)	0.010	0.100
ID	(Intercept)	0.011	0.105
	SpeciePSTR	0.078	0.279

	SpecieSSID	0.088	0.296
Zone	(Intercept)	0.001	0.035
Residual		0.026	0.161

Supplementary Table 19. MG2 Contrast test. Degrees-of-freedom method: kenward-roger. Confidence level used: 0.95

**Contrast**	**Estimate**	**Std. Error**	**t.ratio**	**p.value**
Specie= OFAV				
Contrast dead-live	0.030	0.123	0.319	0.815
Specie=PSTR				
Contrast dead-live	-0.430	0.131	-3.290	0.008
Specie=SSID				
Contrast dead-live	-0.284	0.133	-2.134	0.059

References

1. Grange, J. S., Rybarczyk, H., & Tribollet, A. The three steps of the carbonate biogenic dissolution process by microborers in coral reefs (New Caledonia). Environmental Science and Pollution Research, *22*(18), 13625-13637 (2015).
2. Leggat, W. P., Camp, E. F., Suggett, D. J., Heron, S. F., Fordyce, A. J., Gardner, S., & Ainsworth, T. D. Rapid coral decay is associated with marine heatwave mortality events on reefs. Current Biology, *29*(16), 2723-2730 (2019).
3. Enochs, I. C., Manzello, D. P., Kolodziej, G., Noonan, S. H., Valentino, L., & Fabricius, K. E. Enhanced macroboring and depressed calcification drive net dissolution at high-CO_2 coral reefs. Proceedings of the Royal Society B: Biological Sciences, *283*(1842), 20161742 (2016).
4. Enochs, I. C., Toth, L. T., Kirkland, A., Manzello, D. P., Kolodziej, G., Morris, J. T., ... & Aronson, R. B. Upwelling and the persistence of coral-reef frameworks in the eastern tropical Pacific. Ecological Monographs, *91*(4), e01482. (2021).
5. Enochs, I. C., Manzello, D. P., Tribollet, A., Valentino, L., Kolodziej, G., Donham, E. M., & Price, N. N. Elevated colonization of microborers at a volcanically acidified coral reef. *PLoS One*, *11*(7), e0159818 (2016).
6. Reyes-Nivia, C., Diaz-Pulido, G., Kline, D., Guldberg, O. H., & Dove, S. Ocean acidification and warming scenarios increase microbioerosion of coral skeletons. Global Change Biology, *19*(6), 1919-1929 (2013).
7. Tribollet, A., & Golubic, S. Cross-shelf differences in the pattern and pace of bioerosion of experimental carbonate substrates exposed for 3 years on the northern Great Barrier Reef, Australia. Coral reefs, *24*(3), 422-434. (2005).
8. Tribollet, A., Decherf, G., Hutchings, P., and Peyrot-Clausade, M. Large-scale spatial variability in bioerosion of experimental coral substrates on the Great Barrier Reef (Australia): importance of microborers. Coral Reefs 21, 424-432 (2002).
9. Chazottes, V., Le Campion-Alsumard, T., & Peyrot-Clausade, M. Bioerosion rates on coral reefs: interactions between macroborers, microborers and grazers (Moorea, French Polynesia). Palaeogeography, Palaeoclimatology, Palaeoecology, 113(2-4), 189-198 (1995).
10. Mallela, J., & Perry, C. T. Calcium carbonate budgets for two coral reefs affected by different terrestrial runoff regimes, Rio Bueno, Jamaica. Coral reefs, 26, 129-145 (2007).
11. Vogel, K., Gektidis, M., Golubic, S., Kiene, W.E. & Radtke, G. Experimental studies on microbial bioerosion at Lee Stocking Island, Bahamas and One Tree Island, Great Barrier Reef, Australia: Implications for paleoecological reconstructions. Lethaia 33, 190–204 (2000).
12. Roik, A., Röthig, T., Pogoreutz, C., Saderne, V. & Voolstra, C.R. Coral reef carbonate budgets and ecological drivers in the central Red Sea – A naturally high temperature and high total alkalinity environment. Biogeosciences 15, 6277–6296 (2018).
13. Brown, K. T., Bender-Champ, D., Achlatis, M., van Der Zande, R. M., Kubicek, A., Martin, S. B., & Hoegh-Guldberg, O. Habitat-specific biogenic production and erosion influences net framework and sediment coral reef carbonate budgets. *Limnology and Oceanography*, *66*(2), 349-365. (2021).
14. Cerdeira-Estrada, S., et al. Scale 1:4; Comisión Nacional para el Conocimiento y Uso de la Biodiversidad (CONABIO): Mexico City, Mexico (2018)
15. Dickson, A. G., Sabine, C. L., & Christian, J. R.. Guide to best practices for ocean CO_2 measurements. North Pacific Marine Science Organization (2007)
16. Lewis, E., D. Wallace, and L. J. Allison. Program developed for CO_2 system calculations. Carbon Dioxide Information Analysis Center, managed by Lockheed Martin Energy Research Corporation for the US Department of Energy Tennessee. (1998).
