# Supplementary material for: Newly deceased Caribbean reef-building corals experience rapid carbonate loss and colonization by endolithic organisms
Source: Commun Biol. 2023 Sep 12;6:934. doi: 10.1038/s42003-023-05301-3 (PMC10497637; doi:10.1038/s42003-023-05301-3)
Supplement: Supplementary file 3 — Reporting Summary [file 42003_2023_5301_MOESM3_ESM.pdf]

nature portfolio

Corresponding author(s): Francisco Medellín Maldonado (COMMSBIO-22-2542B)

Last updated by author(s): 08/08/2023

Reporting Summary

Nature Portfolio wishes to improve the reproducibility of the work that we publish. This form provides structure for consistency and transparency in reporting. For further information on Nature Portfolio policies, see our Editorial Policies and the Editorial Policy Checklist.

Statistics

For all statistical analyses, confirm that the following items are present in the figure legend, table legend, main text, or Methods section.

n/a	Confirmed	
☐	☒	The exact sample size (*n*) for each experimental group/condition, given as a discrete number and unit of measurement
☐	☒	A statement on whether measurements were taken from distinct samples or whether the same sample was measured repeatedly
☐	☒	The statistical test(s) used AND whether they are one- or two-sided<br>*Only common tests should be described solely by name; describe more complex techniques in the Methods section.*
☐	☒	A description of all covariates tested
☐	☒	A description of any assumptions or corrections, such as tests of normality and adjustment for multiple comparisons
☐	☒	A full description of the statistical parameters including central tendency (e.g. means) or other basic estimates (e.g. regression coefficient) AND variation (e.g. standard deviation) or associated estimates of uncertainty (e.g. confidence intervals)
☐	☒	For null hypothesis testing, the test statistic (e.g. *F*, *t*, *r*) with confidence intervals, effect sizes, degrees of freedom and *P* value noted<br>*Give P values as exact values whenever suitable.*
☒	☐	For Bayesian analysis, information on the choice of priors and Markov chain Monte Carlo settings
☒	☐	For hierarchical and complex designs, identification of the appropriate level for tests and full reporting of outcomes
☒	☐	Estimates of effect sizes (e.g. Cohen's *d*, Pearson's *r*), indicating how they were calculated

Our web collection on statistics for biologists contains articles on many of the points above.

Software and code

Policy information about availability of computer code

Data collection	no software was used for data collection
Data analysis	linear mixed models and analysis of variance were performed in R (v. 3.6.1), using the lme4, DHARMa and lsmeans packages.

For manuscripts utilizing custom algorithms or software that are central to the research but not yet described in published literature, software must be made available to editors and reviewers. We strongly encourage code deposition in a community repository (e.g. GitHub). See the Nature Portfolio guidelines for submitting code & software for further information.

Data

Policy information about availability of data

All manuscripts must include a data availability statement. This statement should provide the following information, where applicable:

- Accession codes, unique identifiers, or web links for publicly available datasets
- A description of any restrictions on data availability
- For clinical datasets or third party data, please ensure that the statement adheres to our policy

All data are available in the main text, the supplementary materials and the next link: https://github.com/FRAME57/Medellin-Maldonado-et-al.-2022

Human research participants

Policy information about studies involving human research participants and Sex and Gender in Research.

Reporting on sex and gender	N/A
Population characteristics	N/A
Recruitment	N/A
Ethics oversight	N/A

Note that full information on the approval of the study protocol must also be provided in the manuscript.

Field-specific reporting

Please select the one below that is the best fit for your research. If you are not sure, read the appropriate sections before making your selection.

☐ Life sciences ☐ Behavioural & social sciences ☒ Ecological, evolutionary & environmental sciences

For a reference copy of the document with all sections, see nature.com/documents/nr-reporting-summary-flat.pdf

Ecological, evolutionary & environmental sciences study design

All studies must disclose on these points even when the disclosure is negative.

Study description	We predicted the loss of CaCO3 associated with the mortality of D. cylindrus, S. siderea, and P. strigosa colonies due to SCTLD in the Puerto Morelos reef system. For this, we used the estimates of skeletal density obtained from live and dead colonies and field surveys. Field data were collected from July 2018 to September 2019 (during the SCTLD outbreak) in six reef sites across the reef system.
Research sample	Entire coral assemblages were surveyed at each site.
Sampling strategy	In order to obtain the density change between live and dead colonies, we obtained and analyzed samples of D. cylindrus from live colonies in 2015 and from the same colonies in 2019 and 2020 after they had already died. In addition, in 2020, we obtained coral core of S. siderea, P. strigosa, and O. faveolata from live colonies and from colonies that had died due to the SCTLD in 2019. For D. cylindrus, we obtained samples from four colonies because it is a rare species throughout the Caribbean. For P. strigosa, S. siderea and O. faveolata, we considerably increased the number of samples to ensure representation of density change and due to increased availability. In 2020 samples were collected from 17, 12, and 10 colonies respectively. At each reef unit, coral assemblages were surveyed in 10 x 1 m transects. Coral communities were surveyed using 10–25 randomly placed belt transects.
Data collection	All sites were surveyed using the Atlantic and Gulf Rapid Reef Assessment protocol. The following information was recorded for each coral colony within each transect: species name, colony size (maximum diameter, diameter perpendicular to the maximum diameter, and height), bleaching percentage, mortality percentage (new, transition, and old), and the presence of SCTLD or other diseases. For this study, we also recorded colonies with 100% mortality that could be attributed to SCTLD (i.e., recent or transient mortality was still evident). Data was collected by the authors of this study.
Timing and spatial scale	Field data were collected from July 2018 to September 2019 (during the SCTLD outbreak) in six reef sites across the Puerto Morelos reef system
Data exclusions	no data were excluded from the analyses
Reproducibility	Use of standard and well-known field and statistical protocols. We provide all code in an easy to follow (https://github.com/FRAME57/Medellin-Maldonado-et-al.-2022). Furthermore, we include the exact version of the software and names of R packages in the main manuscript.
Randomization	For each reef unit, transects were haphazardly allocated in the field.
Blinding	not relevant. The study consisted in surveying corals assemblages before and after a disease.

Did the study involve field work? ☒ Yes ☐ No

Field work, collection and transport

Field conditions	Supplementary Methods provide a description of environmental conditions considered for this study.
Location	The Puerto Morelos reef system is located near the northeastern portion of the Yucatan Peninsula in Mexico. The specific location of each study site is provided in Fig. 1.
Access & import/export	All coral samples were collected under collection procedures detailed in collection permit No. PPF/DGOPA-061/18 given by the National Commission of Aquaculture and Fisheries of Mexico.
Disturbance	Coral samples were collected by scientific divers on SCUBA. For Dendrogyra cylindrus coral samples were collected using hammers and chisels. Fragments obtained from the colonies of D. cylindrus were ~ 10 cm in diameter and 10-20 cm. For Psudodiploria strigosa, Siderastrea siderea, and Orbicella faveolata, coral samples were collected using an underwater drill. Coral cores samples obtained from the other three species were 3 cm in diameter and 5-15 cm in length. The sections of the live colonies, where sampled, were covered to accelerate healing.

Reporting for specific materials, systems and methods

We require information from authors about some types of materials, experimental systems and methods used in many studies. Here, indicate whether each material, system or method listed is relevant to your study. If you are not sure if a list item applies to your research, read the appropriate section before selecting a response.

Materials & experimental systems

n/a	Involved in the study	
☒	☐	Antibodies
☒	☐	Eukaryotic cell lines
☒	☐	Palaeontology and archaeology
☐	☒	Animals and other organisms
☒	☐	Clinical data
☒	☐	Dual use research of concern

Methods

n/a	Involved in the study	
☒	☐	ChIP-seq
☒	☐	Flow cytometry
☒	☐	MRI-based neuroimaging

Animals and other research organisms

Policy information about studies involving animals; ARRIVE guidelines recommended for reporting animal research, and Sex and Gender in Research

Laboratory animals	This study did not involve vertebrate or higher invertebrates laboratory animals
Wild animals	N/A
Reporting on sex	N/A
Field-collected samples	N/A
Ethics oversight	N/A

Note that full information on the approval of the study protocol must also be provided in the manuscript.
